# Molecular subtyping of nasopharyngeal carcinoma (NPC) and a microRNA-based prognostic model for distant metastasis

**DOI:** 10.1186/s12929-018-0417-5

**Published:** 2018-02-19

**Authors:** Lan Zhao, Alvin H. W. Fong, Na Liu, William C. S. Cho

**Affiliations:** 1 Department of Electronic Engineering, City University of Hong Kong, Hong Kong, China; 2 Department of Clinical Oncology, Queen Elizabeth Hospital, Hong Kong, China; 3 State Key Laboratory of Oncology in South China, Collaborative Innovation Center of Cancer Medicine, Sun Yat-sen University Cancer Center, Guangzhou, China

**Keywords:** Nasopharyngeal carcinoma, Molecular subtyping, Consensus clustering, microRNA, Distant metastasis, Cox regression model

## Abstract

**Background:**

Nasopharyngeal carcinoma (NPC) is a highly invasive and metastatic cancer, with diverse molecular characteristics and clinical outcomes. This study aims to dissect the molecular heterogeneity of NPC, followed by the construction of a microRNA (miRNA)-based prognostic model for prediction of distant metastasis.

**Methods:**

We retrieved two NPC datasets: GSE32960 and GSE70970 as training and validation cohorts, respectively. Consensus clustering was employed for cluster discovery, and support vector machine was used to build a classifier. Finally, Cox regression analysis was applied to constructing a prognostic model for predicting risk of distant metastasis.

**Results:**

Three NPC subtypes (immunogenic, classical and mesenchymal) were identified that are molecularly distinct and clinically relevant, of which mesenchymal subtype (~ 36%) is associated with poor prognosis, characterized by suppressing tumor suppressor miRNAs and the activation of epithelial-­mesenchymal transition. Out of the 25 most differentially expressed miRNAs in mesenchymal subtype, miR-142, miR-26a, miR-141 and let-7i have significant prognostic power (*P* < 0.05).

**Conclusions:**

We proposed for the first time that NPC can be stratified into three subtypes. Using a panel of 4 miRNAs, we established a prognostic model that can robustly stratify NPC patients into high- and low- risk groups of distant metastasis.

**Electronic supplementary material:**

The online version of this article (10.1186/s12929-018-0417-5) contains supplementary material, which is available to authorized users.

## Background

Nasopharyngeal carcinoma (NPC) is one of the five major types of head and neck malignant tumor which develops in the epithelial lining of the nasopharynx [1]. NPC differs significantly from other head and neck cancers in its occurrence, causes and treatment strategies. According to the American Cancer Society, NPC is characterized by its unique geographical and racial distribution with the incidence rate of 20 to 30 cases per 100,000 each year in Southeast Asia as compared with less than 1 case per 100,000 in the United States. Several key etiological factors including genetic [2], Epstein-Barr virus (EBV) infection [3] and dietary [4] implicated as the major causes of NPC. Although NPC is highly sensitive to radiotherapy and chemotherapy, local recurrence and distant metastasis are very common, it is estimated that 15% to 60% of patients will develop local recurrence [5, 6], and 30% to 40% of patients will develop distant metastasis within 4 years after primary treatment [7, 8]. Thus identifying patients at high-risk of local and / or distant metastasis would be crucial for personalized treatment of NPC.

Like other malignancies, NPC is not a single disease which is mainly caused by the intra-tumoral heterogeneity, thus the genetic complexity indeed pose a significant challenge to the targeted therapies for NPC. Owing to the heterogeneous character of NPC, it is necessary to classify NPC patients into different groups which corresponding well with their molecular features as well as clinical outcomes. To this end, not only it can help us understand more about the underlying mechanisms of the tumorigenesis of NPC, but also help us to develop subtype specific therapies for NPC patients. Traditional histopathologic classification of cancer has been carried out by pathologists relies on the histologic appearance and morphological features of the tumors, which only partially reflect the heterogeneity character of cancers. However, in reality, tumors with similar morphological appearance may vary in response to therapy and have distinct clinical outcomes [9]. Recent advancements in genome wide molecular profiling have allowed researchers to classify cancers into homogeneous groups with improved diagnosis and prognosis than traditional classification of cancers [10, 11].

MicroRNAs (miRNAs) are a class of highly conserved noncoding, short regulatory RNAs (19–25 nucleotides) cleaved from 70 to 100 nucleotides hairpin pre-miRNA precursors and are negative regulators of gene expression [12]. MiRNAs are involved in diverse biological functions, including development, differentiation, proliferation, apoptosis and cancers [13]. MiRNA expression signatures are informative, which have been shown to be potential new biomarkers for cancer diagnosis, prognosis and therapy prediction [14–16]. Various miRNA-based classifiers have been built to classify breast cancer [17], lung cancer [18], hepatocellular carcinoma [19], colorectal cancer [20], kidney cancer [21] and myeloma [22] into homogeneous groups based on the specific miRNA expression patterns in cancers.

The recently widely used of miRNA arrays has enabled the large scale profiling of miRNAs in NPC [23]. Here, we analyzed two independent datasets (GSE32960 and GSE70970) which consist of a total number of 558 NPC patients with miRNA expression profiles. We employed an unsupervised classification approach to stratify these patients into three molecular and clinical distinct subgroups (immunogenic, classical and mesenchymal). Of which mesenchymal subtype (~ 36%) is characterized by suppressing tumor suppressor miRNAs and activation of epithelial-mesenchymal transition (EMT). Compared with the other two subtypes, patients classified into mesenchymal subtype have a higher risk of metastasis and poorer distant metastasis-free survival (DMFS). While immunogenic subtype accounts for a small portion of the total NPC patients (~ 19%), they were found to have enrichment in RNA binding and immune related gene sets as well as have good clinical outcomes. Finally, classical subtype was found to be enriched in cell cycle related gene sets, and have an intermediate survival compared with other two subtypes. We also classified the 12 commonly used NPC cell lines into the three subtypes, with six classical, five mesenchymal and one immunogenic subtype, which provides a good in-vitro platform for further subtype-specific studies.

Furthermore, out of the 25 most differentially expressed miRNAs in mesenchymal subtype, miR-142, miR-26a, miR-141 and let-7i have significant prognostic power (*P* < 0.05), as determined by univariate Cox regression analysis. We then built a Cox regression model by using the selected 4 miRNAs. This model can be used to separate the NPC patients into high-risk and low-risk groups of distant metastasis. Thus, our study not only provided a new classification system for NPC, but also identified a panel of biomarkers which may have a great potential to be applied in the clinic for predicting the risk of DMFS.

## Methods

### Data curation and pre-processing

We first searched the Gene Expression Omnibus (GEO) database (www.ncbi.nlm.nih.gov/geo) for all available expression data related to NPC. We came across two relevant datasets, one is in the accession number of GSE32960 [24] which contains 312 non-distant-metastatic paraffin-embedded NPC and 18 paraffin-embedded non-cancer nasopharyngitis biopsy samples. All these samples were collected between Jan 16, 2003, and Feb 25, 2006 from the Sun Yat-sen University Cancer Center (Guangzhou, China), and the clinical staging was classified according to the criteria of the American Joint Committee on Cancer Staging Manual (Seventh Edition). Patients median follow-up was 62.1 months (IQR 47.7–71.5) [24]. Another dataset is in the accession number of GSE70970 [25], which in total contains 246 NPC patients from the Princess Margaret Cancer Center (Toronto, Canada). Those 246 tumor samples were collected at two different time periods. We downloaded normalized miRNA expression and clinical data from GEO database and used the ComBat [26] to remove the batch effects in this dataset.

In total, we collected altogether 558 NPC patients for this subtyping study (Table 1). At which, the miRNA expression profiling of 86 stage II patients from GSE32960 was our training (discovery) dataset to build a classification model. This is because that the expression data of early stages patients are less noisy than the late stages (stage III and stage IV) [27]. Expression noise, or unavoidable stochastic fluctuations [28] were increased along tumorigenesis [27]. More specifically, Han et al. [27] studied the changes of expression noise in different human cancers and found that more than 53.7% genes had increased noise in patients with late stage than early stage cancers. This study showed that a noticeable loss of expression control as cancer development and progression. In order to avoid impacts from ambient noise, we had better use early stage patients’ data to build the model. Besides, since stage I patients are associated with good clinical outcomes than other stages, and only account for a very small portion (< 4%) of total patients in GSE32960, thus they were also excluded from the training dataset. The remaining 226 NPC patients from GSE32960 and 246 NPC patients from GSE70970 were used as two independent validation datasets. Only miRNA features common to both datasets were remained for the following analysis.

**Table 1:** Clinical characteristics of patients according to the classifier in the training and validation sets

	Training set (*n* = 86)				Internal validation set (*n* = 226)				External validation set (*n* = 246)			
	NPC1 (*n* = 37)	NPC2 (*n* = 33)	NPC3 (*n* = 16)	P value^*^	NPC1 (*n* = 115)	NPC2 (*n* = 75)	NPC3 (*n* = 36)	P value^*^	NPC1 (*n* = 104)	NPC2 (*n* = 93)	NPC3 (*n* = 49)	P value^*^
Age, years	47.03	47.91	48.81	0.4199	47.09	45.88	45.92	0.8134	50.23	51.63	48.07	0.4408
Sex, male	22 (59%)	27 (82%)	13 (81%)	0.0760	86 (75%)	56 (75%)	29 (80%)	0.757	73 (70%)	66 (71%)	36 (73%)	0.9156
WHO pathological type	0.1541				0.5689				NA			
Undifferentiated non-keratinising	0	1	0		2	0	0		NA	NA	NA	
Differentiated non-keratinising	1	0	2		3	2	0		NA	NA	NA	
Keratinising squamous cell	36	32	14		110	73	36		NA	NA	NA	
T stage	0.7670				0.5842				0.2508			
T1	13	11	7		22	7	6		32	29	14	
T2	24	22	9		14	13	7		16	27	8	
T3	0	0	0		35	26	10		21	19	13	
T4	0	0	0		44	29	13		35	20	13	
N stage	0.2327				0.797				0.9027			
N0	10	7	1		13	8	5		22	16	11	
N1	27	26	15		43	26	11		34	35	14	
N2	0	0	0		37	26	9		37	32	19	
N3	0	0	0		22	15	11		1	1	2	
TNM stage	NA				0.7387				NA			
I	0	0	0		8	2	2		NA	NA	NA	
II	37	33	16		0	0	0		NA	NA	NA	
III	0	0	0		46	32	13		NA	NA	NA	
IV	0	0	0		61	41	21		NA	NA	NA	
Disease-free survival	0.263^a^				0.0363^a^				0.6443^a^			
Relapses or deaths	5 (14%)	9 (27%)	2 (12%)		40 (28%)	44 (39%)	11 (19%)		37 (36%)	35 (38%)	15 (31%)	
5 year	86%	73%	88%		72%	61%	81%		64%	62%	69%	
Distant metastasis-free survival	0.0215^a^				0.0449^a^				0.0476^a^			
Distant metastases	0 (0.0%)	6 (18%)	1 (6.0%)		24 (17%)	34 (30%)	8 (13%)		13 (12%)	19 (20%)	3 (6.0%)	
5-year	100%	82%	94%		83%	70%	87%		88%	80%	94%	
Overall survival	0.6549^a^				0.1708^a^				0.5951^a^			
Deaths	4 (11%)	5 (15%)	1 (6.0%)		32 (23%)	32 (28%)	10 (17%)		29 (28%)	29 (31%)	12 (24%)	
5-year	89%	85%	94%		77%	72%	83%		82%	69%	76%	

Note: * χ2 test

^a^ Log-rank test

### Identification of NPC subtypes

We first selected 300 most variable miRNAs by calculating the median absolute deviation (MAD) of each miRNA across 86 patients from the training dataset, the variable miRNAs were retained and row-normalized expression for the following analysis. Next, we performed consensus clustering [29] consisted of 1000 iterations of hierarchical clustering, with 0.9 subsampling ratio, and agglomerative average linkage and Pearson correlation to cluster these 86 patients. We used the gap statistic [30], which is a measure of within-cluster dispersion to assess the optimal number of clusters. Silhouette width was computed to identify the most representative samples within each cluster. Finally, we retained samples with positive silhouette width (*n* = 77) to build a classifier for NPC.

### Cell culture

Human NPC cell line C17 was obtained through the generosity of Dr. Pierre Busson (Institut Gustave Roussy, France) and cultured in RPMI 1640 medium supplemented with 7.5% fetal bovine serum (FBS), 25 mM HEPES and 7 μM ROCK inhibitor Y-27632. C666, CNE2, HNE1, HK1, HONE1, NP69, NP460 were kindly provided by Prof. George S.W. Tsao (The University of Hong Kong, Hong Kong). C666, CNE2, HK1 and HONE1 were cultured in RPMI 1640 medium supplemented with 10% FBS. HNE1 was cultured in cultured in DMEM medium supplemented with 5% FBS and 5% newborn calf serum. NP69 was cultured in Keratinocyte-SFM medium supplemented with 0.05 mg/ml bovine pituitary extract and 5 ng/ml epidermal growth factor. NP460 was cultured in Defined Keratinocyte-SFM medium and EpiLife Medium in 1:1 ratio. HK1-LMP1, HK1-LMP1 Cis R, HONE1-EBV and HONE1-EBV Cis R were gifts from Prof. Brigette B.Y. Ma (The Chinese University of Hong Kong, Hong Kong). These cell lines were cultured as previously described [31]. All cells were maintained at 37 °C and 5% CO_2_ humidified atmosphere. All culture reagents were obtained from Thermo Fisher Scientific. List of the NPC cell lines involved in our study can be found at Table 2.

**Table 2:** NPC cell line classification results

Cell line name	Cell line description	Subtype
C666	Undifferentiated nasopharyngeal carcinoma	Classical
HK1	Well differentiated squamous carcinoma	Classical
HK1LMP1	HK1 with LMP1 transfected	Classical
HK1LMP1CisR	HK1-LMP1 with cisplatin resistance	Classical
HONE1EBVCisR	Poorly differentiated squamous carcinoma	Classical
NP69	Immortalized nasopharyngeal-derived epithelial cells	Classical
C17	EBV-positive metastatic NPC	Mesenchymal
CNE2	Poorly differentiated squamous carcinoma	Mesenchymal
HNE1	Poorly differentiated squamous carcinoma	Mesenchymal
HONE1	HONE1 with EBV infected	Mesenchymal
HONE1EBV	HONE-1-EBV with cisplatin resistance	Mesenchymal
NP460	Immortalized nasopharyngeal-derived epithelial cells	Immunogenic

### miRNA isolation and quantitative RT-PCR

Total RNA containing miRNA were extracted from cell lines using miRNeasy Mini Kit (QIAGEN, USA), and DNase I digestion were performed according to the manufacturer’s instructions. Total RNA was eluted in 30 μL RNase-free water. RNA concentration was determined by NanoDrop One spectrophotometer (Thermo Fisher Scientific, USA). cDNA was reverse transcribed from 1 μg of total RNA using miScript II RT Kit (QIAGEN, USA). qPCR was carried out with miScript SYBR Green PCR Kit (QIAGEN, USA) on the LightCycler 480 System (Roche, Switzerland). hsa-miR-26a, hsa-miR-29b, hsa-miR-200b, hsa-miR-370, hsa-miR-622, hsa-miR-1248, hsa-miR-1293, hsa-miR-2053, hsa-let-7d and hsa-let-7 were predesigned primers (miScript Primer Assays MS00029239, MS00009289, MS00009023, MS00045885, MS00005117, MS00014238, MS00014539, MS00044569, MS00003136 and MS00006489, QIAGEN, USA). Amplification reactions were done in triplicate for each examined sample. RNU6 snRNA (miScript Primer Assay MS00033740, QIAGEN, USA) served as the endogenous control for normalization. Cycling conditions were 95 °C for 15 min, followed by 45 cycles at 94 °C for 15 s, 55 °C for 30 s and 70 °C for 30 s. Relative quantification of target miRNA expression was calculated using the 2 ^-ΔΔCt^ method.

### Generation of the NPC classifier and classification

To build the NPC classifier, we also did a feature (miRNA) selection process which involved two filtering steps to select the most representative and predictive miRNAs. First, we used the Significance Analysis of Microarrays (SAM) algorithm (R package siggenes version 1.42.0) to identify miRNAs significantly differentially expressed (false discovery rate (FDR) < 0.01) between each subtype and the other two. Next, we calculated the Area Under the Curve (AUC) (R package ROCR version 1.0–7) to assess each miRNA’s ability to separate two clusters. The retained 10 miRNA with AUC > 0.9 were trained by Support Vector Machine (SVM) to build a classifier. The expression profiles of the two validation datasets, and NPC cell line data were mean or median centered across all samples and then subjected to classification using the classifier built based on the training dataset.

### miRNA target prediction and gene set enrichment analysis (GSEA)

Differentially expressed miRNAs between each subtype were identified by using the R package limma [32], with absolute log2 fold change greater than 1 and Benjamini-Hochberg-adjusted *p*-value less than 0.05. We then obtained experimentally validated target genes of each differential miRNA based on the miRWalk 2.0 database (http://zmf.umm.uni-heidelberg.de/apps/zmf/mirwalk2/) [33]. GSEA is a widely used method to interpret expression data at the level of gene sets, that is, groups of genes that share common biological function, or regulation [34]. In this study, GSEA with annotated gene sets from KEGG, Reactome and Gene Ontology (GO) was done with Enrichr tool (http://amp.pharm.mssm.edu/Enrichr/) [35].

### Survival analysis

In the two datasets, DMFS were calculated from treatment to the date to the first distant relapse, and disease-free survival (DFS) to the first relapse at any site or death from any cause, whichever occurred first, and overall survival (OS) to death from any cause [24, 25]. Survival analysis was performed using the Kaplan-Meier method, and the differences in time to an event (death or recurrence) between curves were assessed by using the log-rank tests. Adjusted *P* values were obtained by Benjamini and Hochberg’s method of less than 0.05 were considered to be statistically significant.

### Cox regression model

In order to identify a miRNA signature associated with risk of distant metastasis (DM), we did a differential miRNA expression analysis between mesenchymal subtype and non-mesenchymal subtypes. In total, we identified 25 differentially expressed miRNAs (Table 3) (limma package [36] in R) with a cutoff of absolute log2 fold change greater than 1 and adjusted *P* value less than 0.05. Among the 25 miRNAs, miR-142, miR-26a, miR-141 and let-7i have significant prognostic power (*P* < 0.05) (Table 3), as determined by univariate Cox regression analysis. For identification of high-risk distant metastasis, we built a multivariate Cox regression model using the selected 4 miRNAs.

**Table 3:** Differentially expressed miRNAs in mesenchymal subtype

	Limma analysis		Cox regression analysis	
ID	logFC	adj.P.Val	Hazard ratio (95% CI)	*P* value
ebv-miR-BART11-5p	−1.54706	2.16E-25	0.9777 (0.8187242–1.167488)	0.803
hsa-let-7a	−1.40183	8.66E-25	0.963 (0.7880834–1.176745)	0.711
hsa-let-7b	− 1.06179	2.16E-25	0.9471 (0.7233448–1.239976)	0.691
hsa-let-7d	−1.23859	2.46E-62	0.789 (0.5750843–1.081624)	0.141
hsa-let-7f	−1.1923	2.45E-57	0.759 (0.5521583–1.042009)	0.0867
hsa-let-7i	−1.3304	1.87E-56	0.735 (0.5547504–0.9745582)	0.0329^*^
hsa-miR-103	−1.21743	1.79E-43	0.87 (0.6585442–1.148594)	0.328
hsa-miR-1246	−1.08753	3.66E-27	0.857 (0.6564593–1.117855)	0.251
hsa-miR-1248	−1.34498	1.76E-38	0.89 (0.6972987–1.136929)	0.352
hsa-miR-1308	−1.21742	6.61E-23	0.98 (0.7887421–1.218035)	0.857
hsa-miR-141	−1.16218	1.26E-28	0.752 (0.5827463–0.9715151)	0.0291^*^
hsa-miR-142-3p	−1.0139	3.21E-30	0.55 (0.397994–0.7602394)	0.000166^*^
hsa-miR-16	−1.12108	1.17E-16	0.879 (0.7166531–1.078863)	0.217
hsa-miR-1973	−1.22079	3.55E-19	1.0439 (0.8511332–1.280275)	0.678
hsa-miR-1975	−1.03043	1.01E-11	1.0092 (0.8323964–1.223649)	0.925
hsa-miR-19b	−1.02683	1.09E-30	0.902 (0.6693412–1.216764)	0.5
hsa-miR-200b	−1.08776	1.26E-28	0.799 (0.6070927–1.050777)	0.106
hsa-miR-21	−1.67241	7.01E-37	0.99486 (0.820039–1.206952)	0.958
hsa-miR-23a	−1.19406	1.13E-46	0.796 (0.5968523–1.06213)	0.124
hsa-miR-24	−1.09488	6.93E-31	0.9481 (0.7189929–1.250106)	0.706
hsa-miR-26a	−1.48288	8.51E-44	0.656 (0.5161167–0.8343482)	0.000469^*^
hsa-miR-29a	−1.20827	8.57E-23	0.829 (0.670324–1.024992)	0.0857
hsa-miR-615-3p	1.051951	6.30E-36	1.113 (0.8244393–1.502931)	0.486
hsa-miR-767-5p	1.179607	1.24E-13	1.0842 (0.8985411–1.308245)	0.387
hsa-miR-922	1.154044	7.59E-12	0.9802 (0.8252847–1.164265)	0.819

Note: ^*^ Significant difference *P* < 0.05

logFC: log2 fold change; adj.P.Val: Benjamini-Hochberg-adjusted p-value

## Results

### Unsupervised clustering identifies three subtypes in NPC

**Fig. 1:**
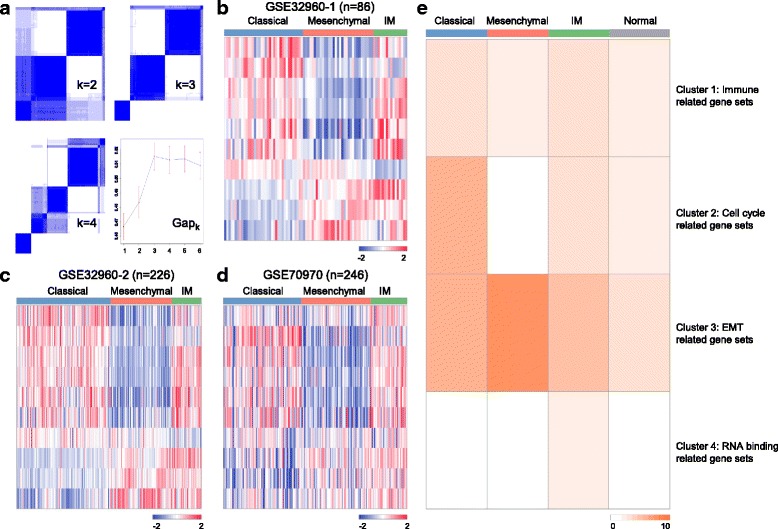
Unsupervised classification identified three molecular distinct subtypes of nasopharyngeal carcinoma. **a** Unsupervised classification of the training dataset shows the optimal cluster number is three. A classifier was constructed (using 10 unique miRNAs) to categorize patients in each of the subtypes; (**b-d**) The training dataset (86 patients), GSE32960 set (226 patients) and GSE70970 set (246 patients) were classified into three subtypes according to the classifier, respectively. In the heatmaps, columns correspond to patients, and rows to 10 miRNAs (miR-1248, miR-29b, let-7f, let-7d, miR-26a, miR-200b, miR-370, miR-2053, miR-1293 and miR-622). Expression values are represented by different colors, red means higher expression values, and green for lower expression values. Note: IM is short for immunogenic; (**e**) A *p*-value heatmap to represent NPC subtype enriched pathways (normal group was used as control), values in the heatmap equal to -log10 (p-value)

Unsupervised clustering was applied to the 86 stage II NPC patients from the GSE32960 dataset, which revealed 2 to 4 well-defined clusters (Fig. 1a). GAP statistics were calculated to determine the optimal number of clusters, and a peak was found at k = 3 (Fig. 1a). Silhouette width analysis was subsequently performed to select the most coherent samples within each cluster. The average silhouette width was 0.22 (range from 0.17 to 0.38), indicating the robustness of the classification. A total number of 77 samples (~ 90%) with positive silhouette width were retained to build the classifier. Next, we selected 10 most predictive miRNAs (miR-622, miR-29b, miR-1293, miR-1248, miR-26a, let-7d, miR-200b, let-7f, miR-2053 and miR-370) as features to build a SVM classifier. The classifier can be used to classify the 86 NPC patients into three subtypes: NPC1 (37 patients, 43%), NPC2 (33 patients, 38%) and NPC3 (16 patients, 19%) (Table 1).

### Classification in the validation and cell line datasets

In order to investigate whether these three subtypes exist in other datasets, we first performed classifications in the two validation datasets. One is an internal dataset contains the remaining 226 NPC patients from GSE32960 and another is an external dataset contains 246 NPC patients from GSE70970. Patients in these two datasets can be classified into three subtypes with a similar proportion of patients being distributed among subtypes (Table 1), which may suggest a general inter-tumor heterogeneity pattern exist in the NPC patients. Furthermore, we can also classify the 12 commonly used NPC cell lines into the three subtypes using our classification system, with six classical, five mesenchymal and one immunogenic subtype (Table 2). The cell line classification results may provide a good in vitro platform for studying NPC biology and finally developing subtype-specific therapies for the patients.

### Functional annotation of NPC subtypes

There are distinct miRNA expression patterns between subtypes as observed in the heatmaps (Fig. 1b-d). Among the 10 miRNAs, miR-1248, miR-29b, miR-26a, let-7f and let-7d were specifically down-regulated in NPC2 (mesenchymal subtype); while miR-622 and miR-1293 were specifically down-regulated in NPC1 (classical subtype); and the majority of miRNAs (80%) were up-regulated in NPC3 (immunogenic subtype) compared to the other two subtypes (Fig. 1b-d). We also compared the 10 miRNAs expression patterns between non-cancer (*n* = 18) and cancer groups in the training (*n* = 86) and validation (*n* = 226) datasets. Results show that there exist clearly negative correlations between non-cancer and mesenchymal groups. Specifically, miR-29b, let-7f, let-7d and miR-26a were strikingly more highly-expressed; and miR − 622 was lowly-expressed in the non-cancer group (Additional file 1: Figure S1). The distinct miRNAs expression patterns between patient (especially in the mesenchymal subtype) and normal groups also suggested that these 10 miRNAs are cancer-specific dysregulated miRNAs, and can be investigated further to develop personalized therapies for NPC patients.

To identify the association of biological pathways with subtypes, we subsequently performed GSEA for the enriched target genes in each subtype. In total, we obtained 55 target genes in NPC1, 1, 241 target genes in NPC2, 35 target genes in NPC3 and 251 target genes in non-cancer group (Additional file 2: Table S1) by searching the miRWalk 2.0 database [33]. Gene sets significantly enriched for each subtype were displayed in Additional file 3: Table S2, and in total there were 451 gene sets that significantly enriched (adjusted *P* < 0.05) in at least one NPC subtype (Additional file 3: Table S2). We then used the k-means clustering method with k = 4 to cluster these 451 gene sets, and a *P* value heatmap was built to show the gene sets enriched in each subgroup (Fig. 1e). EMT and metastasis related gene sets were most highly enriched in NPC2, thus we named this group of patients as mesenchymal subtype. Cell cycle related gene sets were specifically enriched in NPC1, which reflect a typical characteristic of the rapidly proliferating tumor cells, therefore we name this subtype as classical. Various RNA binding and immune related gene sets were most enriched in NPC3. Although RNA binding related gene sets are specifically enriched in NPC3 (Fig. 1e), the biological functions of these gene sets are still not fully understood, so we named NPC3 as immunogenic (Fig. 1e).

### Clinical characterization of NPC subtypes

**Fig. 2:**
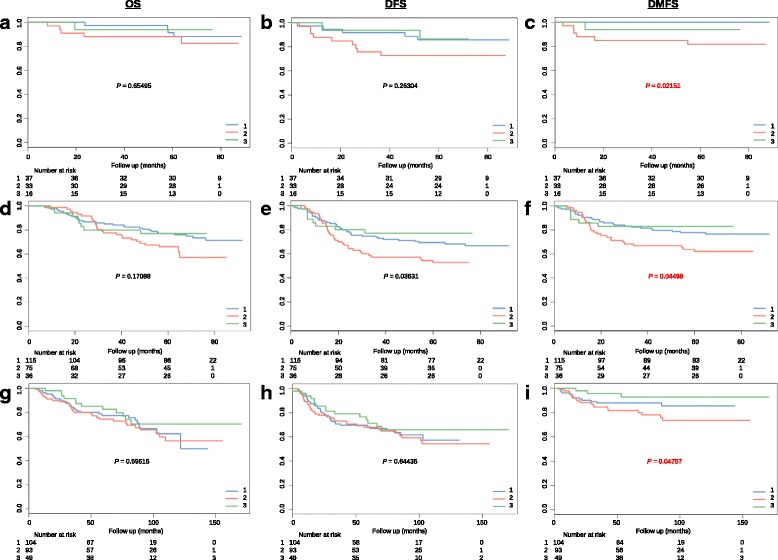
Mesenchymal subtype have poor prognosis compared with other two subtypes. **a-c** Kaplan-Meier graphs depicting overall survival (OS), disease-free survival (DFS) and distant metastasis (DMFS) within the training data set (86 patients) stratified by the NPC classification, and *p* values are based on log-rank tests; (**d-f**) Kaplan-Meier graphs depicting OS, DFS and DMFS within the GSE32960 set (226 patients) stratified by the subtype classifications; (**g-i**) Kaplan-Meier graphs depicting OS, DFS and DMFS within the GSE70970 set (246 patients) stratified by the subtype classifications

Survival analysis by Kaplan-Meier for each subtype indicated that mesenchymal subtype had the worst clinical outcomes (significantly poorer DMFS) compared with the classical and immunogenic subtypes (Fig. 2c, f, i). There was no significant differences among the three subtypes for other clinical endpoints such as OS and DFS (Fig. 2 a-b, d-e and g-h), which suggested that these subtypes only have DMFS differences both in training and validation datasets.

The average age of the external validation dataset is slightly older than the training dataset, and the ratio of male patients in each subtype vary from 59% to 81%. The detailed clinical information of these subtypes were summarized in the Table 1. We also investigated the association among the subtypes with other clinical factors, such as age, sex and tumor stage, which revealed no significant differences (Table 1). This analysis demonstrated that other clinical factors cannot predict DMFS, and supports the use of subtypes as a reliable prognostic factor in NPC.

### Cox proportional hazards model can separate the NPC patients into high-risk and low-risk groups of distant metastasis

**Fig. 3:**
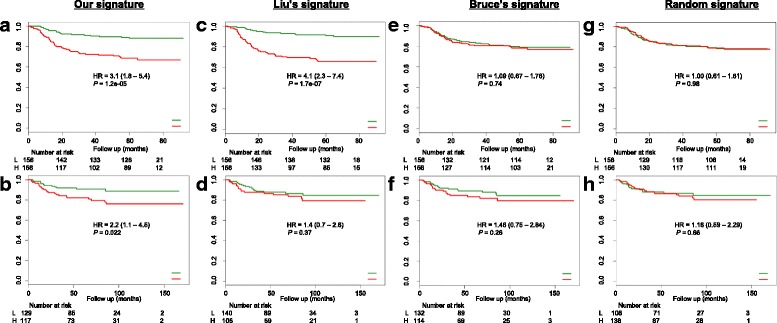
Cox model can separate NPC into high- and low- risk of distant metastasis groups. **a-b** Cox model built by using our signature (4 miRNAs: miR-142, miR-26a, miR-141 and let-7i) can separate NPC into high- and low- risk groups of distant metastasis; (**c-d**) Cox model built by using Liu’s signature (5 miRNAs: miR-93, miR-26a, miR-142, miR-29c and miR-30e) and performances; (**e-f**) Cox model built by using Bruce’s signature (4 miRNAs: miR-154, miR-449b, miR-140 and miR-34c) and performances; (**g-h**) Cox model built by using randomly generated signature (4 miRNAs: miR-653, miR-766, miR-1302 and miR-505) and performances

The Cox proportional hazards model is one of the most popular used method to analyze survival data [37]. Out of the 25 most differentially expressed miRNAs in mesenchymal subtype, miR-142, miR-26a, miR-141 and let-7i have significant prognostic power (*P* < 0.05), as determined by univariate Cox regression analysis (Table 3). For identification of high-risk distant metastasis, we built a multivariate Cox regression model using the selected 4 miRNAs. We calculated the risk scores based on the model for each patients in the training dataset (*n* = 312), a cutoff was determined by the median risk score (0.027), and patients were classified into high-risk (> 0.027) and low-risk (< 0.027) groups. Survival analysis were subsequently performed to investigate if there were survival differences between these two groups, compared with patients with low-risk scores, patients with high risk scores in the training dataset had shorter DMFS (hazard ratio [HR] 3.1, 95% CI 1.8–5.4; *P* = 1.2e-05), and validation dataset DMFS (2.2, 1.1–4.5; *P* = 0.022) (Fig. 3a-b).

We also investigated if other miRNA signatures, such as Liu’s 5-miRNA signature (miR-93, miR-26a, miR-142, miR-29c and miR-30e) [24], Bruce’s 4-miRNA signature (miR-154, miR-449b, miR-140 and miR-34c) [25] and randomly generated 4-miRNA signature (miR-653, miR-766, miR-1302 and miR-505) were significantly associated with DMFS. Although Liu’s 5-miRNA signature has significant prognostic power in the training dataset (*P* = 1.7e-07), it performed worse with its *p*-value of 0.37 in the validation dataset (Fig. 3c-d). Bruce’s and randomly generated 4-miRNA signatures all received poor performances both in training and validation datasets (Fig. 3e-f, and g-h).

## Discussion

Like other cancer types, not all NPC patients will present identical clinical outcomes after treatment, some will result in relatively good treatment outcomes, whereas some are not. The major reason for such phenomenon is caused by the intra-tumoral heterogeneity. How to classify and select the right treatment strategies for NPC patients become crucial tasks. Recent genome wide molecular profiling provide an opportunity to investigate the genetic changes during the development and progression of cancers, and have been widely used in the cancer classification studies [38, 39]. More and more genome wide molecular profiling studies have been carried out in NPC, and there are some gene expression datasets available for NPC, however, number of patients in each cohort is limited.

Unlike mRNA, miRNAs are short noncoding RNA and are negative regulators of gene expression. MiRNAs are involved in cancer by functioning as tumour suppressors or oncogenes. MiRNA expression signatures are informative and have been successfully used as diagnostic and prognostic markers for various types of cancers [17–22]. We found two big miRNA expression datasets containing a total number of 550 NPC patients [24, 25], and thus employed them in our subtyping study. According to the literature search, Liu et al. [24] (GSE32960) dataset contains the largest NPC miRNA profiling data (312 tumor and 18 normal control) so far, while Bruce et al. [25] (GSE70970) dataset contains 246 NPC patients. More specifically, we used 86 stage II patients from GSE32960 as our training dataset, the remaining patients from GSE32960 and GSE70970 were used as two validation datasets. We identified three subtypes in NPC: classical, mesenchymal and immunogenic subtypes. We found that patients classified into mesenchymal subtype tend to have the worst clinical outcomes, thus we put our emphasis on this subtype.

Mesenchymal subtype-specific miRNAs, such as let-7 family, miR-29b, miR-29a, and miR-26a, are the major contributor to the poor prognosis of the mesenchymal subtype. The let-7 family of miRNAs contain several members: let-7 (−a, −b, −c, −d, −e, −f, −g, and -i), they are highly conserved across animal species [40], and are widely considered as tumor suppressor miRNAs. Let-7 miRNAs are frequently downregulated in various types of cancers, including in NPC [41–43]. Wong et al. [42] found that let-7 expression were downregulated in NPC cells compared with normal nasopharyngeal cells, and let-7 can inhibit cell proliferation through renal cell down-regulation of c-Myc expression. Li et al. [43] investigated miRNA expression at different stages of NPC tissue samples and found that different members from let-7 family were dysregulated from early stage to the late stage. In our study, we found that let-7 regulate much more EMT and migration related genes than other miRNAs, indicating it plays a critical role in the poor prognosis characteristic of mesenchymal subtype. Other mesenchymal subtype specific miRNAs include miR-29b, miR-29a, and miR-26a. The miR-29 family consists of three members: miR-29a, miR-29b, and miR-29c, differing only in few bases in the 3′ end nucleotides, among them miR-29b is the most highly expressed member [44]. The miR-29 family functioning as a tumor suppressor in many types of cancers, which can regulate apoptosis, cell proliferation and differentiation. MiR-29b can regulate the expression of tumor suppressor p53, and is recognized as an important regulator of EMT [45]. Reduced expression of miR-29c has been reported in several NPC studies [24, 46]. In our study, we found that miR-29a and miR-29b were the mesenchymal subtype specific miRNAs, and they were significantly downregulated in mesenchymal subtype. Interestingly, we also found that miR-29a was in our Cox model associated with DMFS. MiR-26a has two precursors: miR-26a-1 and miR-26a-2, which located in chromosomes 3 and 12, respectively. MiR-26 is down-regulated in other cancers as well as in NPC [24]. Ma et al. [47] found that miR-26a can inhibit the EMT by down regulation of EZH2 expression, Liang et al. [48] found that miR-26a can regulate the biogenesis of let-7d, and Slaby et al. [49] had proved that miR-26a was associated with tumor relapse in renal cell carcinoma, which all corresponds well with the expression pattern of miR-26a in our study. We also identified miR-1248, which has not been reported to be associated with EMT in NPC.

In the era of precision oncology, molecular subtyping of NPC is important. Not only it can stratify patients into different subgroups, but also may help in triaging treatment strategies for the patients in different subgroups. In our study, we identified some targets for mesenchymal subtype, which might have implication with clinical values. In the meantime, we found that mesenchymal subtype patients have enriched for EMT and /or migration related miRNAs and pathways, thus may account for the worst clinical outcomes of the mesenchymal subtype. Compared to mesenchymal subtype, classical subtype have better clinical outcome, and the majority of patients (~ 42%) are classified into classical subtype. Finally, we identified four prognostic miRNAs (miR-142, miR-26a, miR-141 and let-7i) and build a Cox regression model. The model can be used to separate the NPC patients into high- and low-risk groups of distant metastasis. Among the four miRNAs, miR-142 and miR-26a have been reported by Liu et al. [24] as prognostic factors for DFS in NPC, which indicate that these two miRNAs can be used to predict the risk of both DFS and DMFS. As one member of the miR-200 family, miR-141 was reported to be dysregulated in many cancers, participating in various cellular processes including EMT, cell proliferation and migration [50]. MiR-141 expression has been proved to be negatively correlated with survival in NPC [51]. In summary, the 4-miRNA Cox model is strongly associated with NPC tumorigenesis, and has been demonstrated to be prognostic signature of DMFS in our study.

To our best knowledge, this is the first study to classify the NPC patients into three molecular and clinical distinct subtypes based on miRNA expression profiles. We also classified the 12 commonly used NPC cell lines into the three subtypes, which can provide in vitro platforms to study subtypes of NPC. The present findings warrant, larger patients datasets validation before applied into the clinic.

## Conclusions

We proposed for the first time that NPC can be stratified into three subtypes. Using a panel of 4 miRNAs, we established a prognostic model that can robustly stratify NPC patients into high- and low- risk groups of distant metastasis.

## Additional files

Additional file 1: **Figure S1.** Ten-miRNA expression patterns in the training (86 NPC and 18 normal) and validation (226 NPC and 18 normal) datasets. In the heatmaps, columns correspond to samples, and rows to the 10 miRNAs. Expression values are represented by different colors, red means higher expression values, and green for lower expression values. Note: IM is short for immunogenic. (PDF 53 kb)

Additional file 2: **Table S1.** Subtype target gene list. (XLSX 30 kb)

Additional file 3: **Table S2.** Subtype enriched pathways. (XLSX 136 kb)

## Abbreviations

AJCC: American Joint Committee on Cancer
AUC: Area under the curve
DFS: Disease-free survival
DMFS: Distant metastasis-free survival
EMT: Epithelial-mesenchymal transition
GSEA: Gene set enrichment analysis
HR: Hazard ratio
MAD: Median absolute deviation
NPC: Nasopharyngeal carcinoma
OS: Overall survival
SAM: Significance analysis of microarrays
SVM: Support vector machine
