# Supplementary figures and images for: Molecular subtyping of nasopharyngeal carcinoma (NPC) and a microRNA-based prognostic model for distant metastasis
Source: J Biomed Sci. 2018 Feb 19;25:16. doi: 10.1186/s12929-018-0417-5 (PMC5817810; doi:10.1186/s12929-018-0417-5)

a

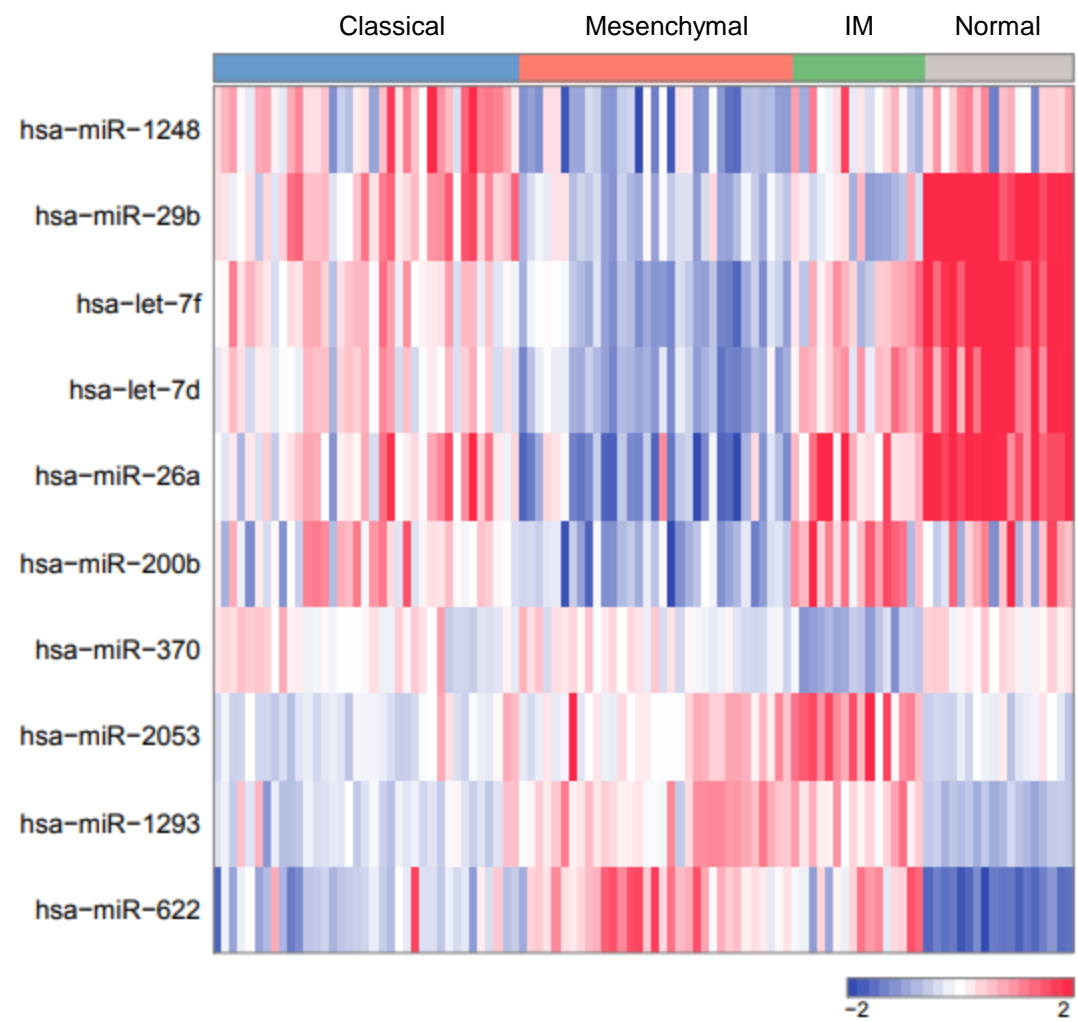

b

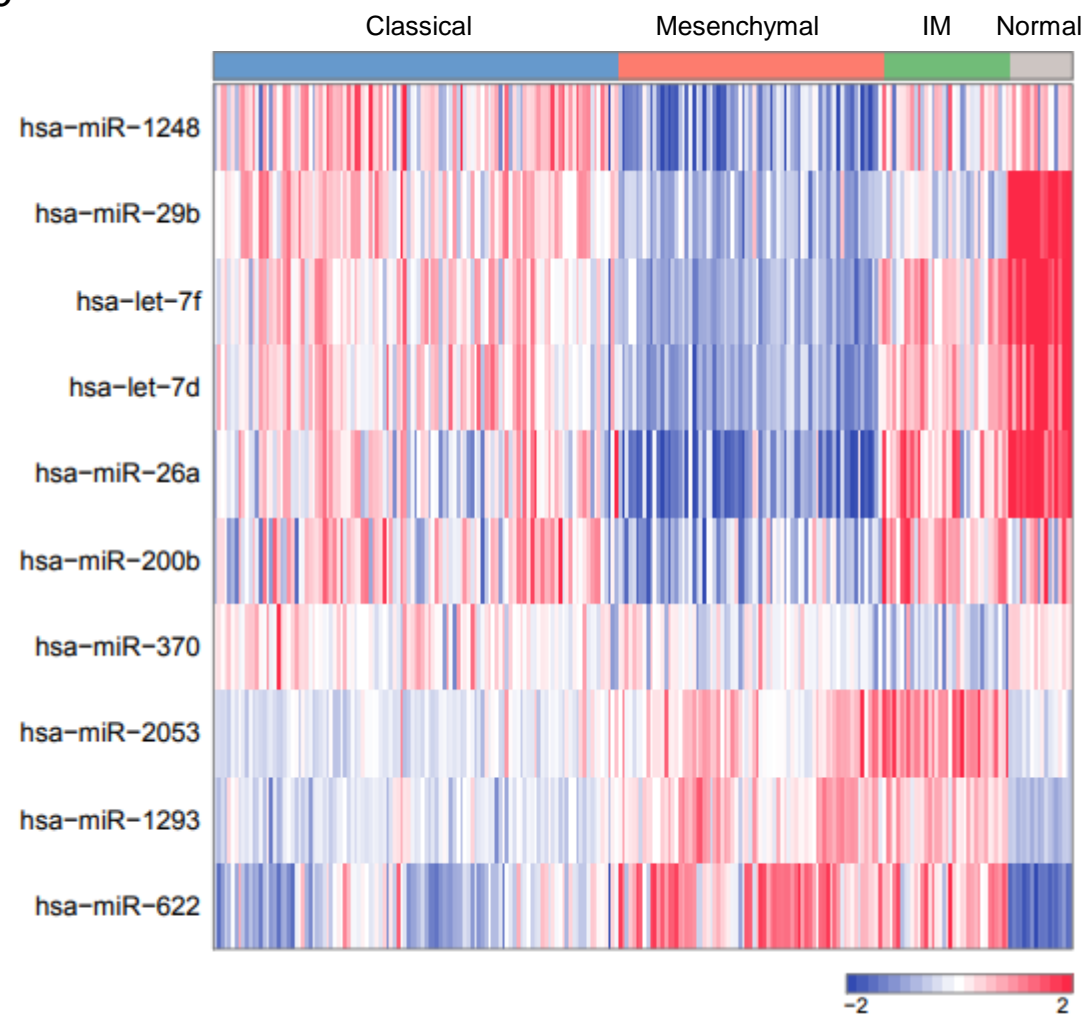

Supplement: Supplementary file 1 — Figure S1. Ten-miRNA expression patterns in the training (86 NPC and 18 normal) and validation (226 NPC and 18 normal) datasets. In the heatmaps, columns correspond to samples, and rows to the 10 miRNAs. Expression values are represented by different colors, red means higher expression values, and green for lower expression values. Note: IM is short for immunogenic. (PDF 53 kb) [file 12929_2018_417_MOESM1_ESM.pdf]
